# Supplementary material for: Cardiolipin-Containing Lipid Membranes Attract the Bacterial Cell Division Protein DivIVA
Source: Int J Mol Sci. 2021 Aug 3;22(15):8350. doi: 10.3390/ijms22158350 (PMC8348161; doi:10.3390/ijms22158350)
Supplement: Supplementary file 1 [file ijms-22-08350-s001.zip › ijms-1305548-supplementary.pdf]

SUPPLEMENTARY INFORMATION

The cell division protein DivIVA binds preferentially to cardiolipin-containing lipid membranes

Naďa Labajová[1#], Natalia Baranova[2], Miroslav Jurásek[3], Robert Vácha[3#], Martin Loose[2], and Imrich Barák[1#]

[1] Institute of Molecular Biology SAS, Dubravska cesta 21, 845 51 Bratislava, Slovakia

[2] Institute of Science and Technology Austria (IST Austria), Am Campus 1, 3400 Klosterneuburg, Austria

[3] CEITEC and Faculty of Science, Masaryk University, Kamenice 5, Brno, 625 00, Czech Republic

[#] For correspondence: umbinapa@savba.sk, imrich.barak@savba.sk or robertvacha@gmail.com

Table S1 Strains and plasmids

Strain	Description	Reference
E. coli MM294	*lnV44(AS) rfbC1 endA1 spoT1 thi-1 hsdR17 creC510*	[1]
E. coli DH5α	*ΔlacZ ΔM15 Δ(lacZYA-argF) U169 recA1 endA1 hsdR17(rK-mK+) supE44 thi-1 gyrA96 relA1*	[2]
E. coli BL21(DE3)pLysS	*ompT, hsdSB(rB-rB-), dcm, gal pLysS*	[3]
**Plasmid**		
pET-26b		Novagen
pTB146	bla $lacI^q$ P_{T7}::h-sumo	[4]
pET26-$divIVA_{Cd}$	full length *$divIVA_{Cd}$* gene NdeI/BamHI inserted in pET-26b	this study
pTB-$divIVA_{Cd}$	full length *$divIVA_{Cd}$* gene SapI/BamHI inserted in pTB146	this study
pET-26b-Δ60-$DivIVA_{Cd}$	first 160 nt lacking *$divIVA_{Cd}$* gene NdeI/BamHI inserted in pET-26b	this study

Table S2 Oligonucleotide list

Oligonucleotid name	Sequence (5'→3')
divIVAcdNdeIS	CGTCGTCGTCATATGCTAACTCCAATTGAGATAG
divIVAcdSapIS	CGTCGTGCTCTTCCGGTATGCTAACTCCAATTGAGATAG
divIVAcdBamHIE	CGTCGTCGTGGATCCGCTTATTCTAAAGTTGTAGCAGC
divIVAcd_del60_NdeIS	GATGATGATCATATGAATATTGAAGAAACACTAAAAG

Table S3 Lipid mixtures

	PC	PG	CL	PS	TF-CL	Rh-PE
**L1**	70.0%	30.0%				
**L2**	100.0%					
**L3**	67.5%	30.0%	2.5%			
**L4**	65.0%	30.0%	5.0%			
**L5**	62.5%	30.0%	7.5%			
**L6**	55.0%	30.0%	15.0%			
**L1***	69.9%	30.0%			0.1%	
**L4***	64.9%	30.0%	5.0%		0.1%	
**L1****	69.9%	30.0%				0.1%
**L4****	64.9%	30.0%	5.0%			0.1%

PC, phosphatidylcholine;
PG, phosphatidylglycerol;
CL, cardiolipin;
Rh-PE, lissamine rhodamine phosphatidylethanolamine;
TF-CL, TopFluor cardiolipin

Table S4 P-values from sedimentation assays with full length DivIVA$_{Cd}$

	no lipids	L1	L2	L4	L6
**L1**	0.0017	X	0.0211	0.0009	<0.0001
**L2**	0.0759	X	X	<0.0001	<0.0001
**L4**	<0.0001	X	X	X	<0.0001
**L6**	<0.0001	X	X	X	X

Table S5 P-values from sedimentation assays with truncated Δ60-DivIVA$_{Cd}$

	no lipids	L1	L2	L4	L6
**L1**	0.0777	X	0.0228	0.2373	0.3302
**L2**	0.3355	X	X	0.1416	0.2035
**L4**	0.4835	X	X	X	0.9432
**L6**	0.5150	X	X	X	X

Table S6 Summary of values obtained in sedimentation assays

	$DivIVA_{Cd}$		
	**SOL (%)**	**PEL (%)**	**STDEV (%)**
**no SUVs**	95.4	4.6	5.3
**L1**	89.9	10.1	4.9
**L2**	94.7	5.3	2.8
**L4**	80.9	19.1	4.6
**L6**	51.5	48.5	7.1
	$\Delta 60\text{-}DivIVA_{Cd}$		
	**SOL (%)**	**PEL (%)**	**STDEV (%)**
**no SUVs**	92.9	7.1	4.6
**L1**	90.0	10.0	3.4
**L2**	93.5	6.5	5.6
**L4**	92.4	7.6	4.1
**L6**	90.4	9.6	4.6

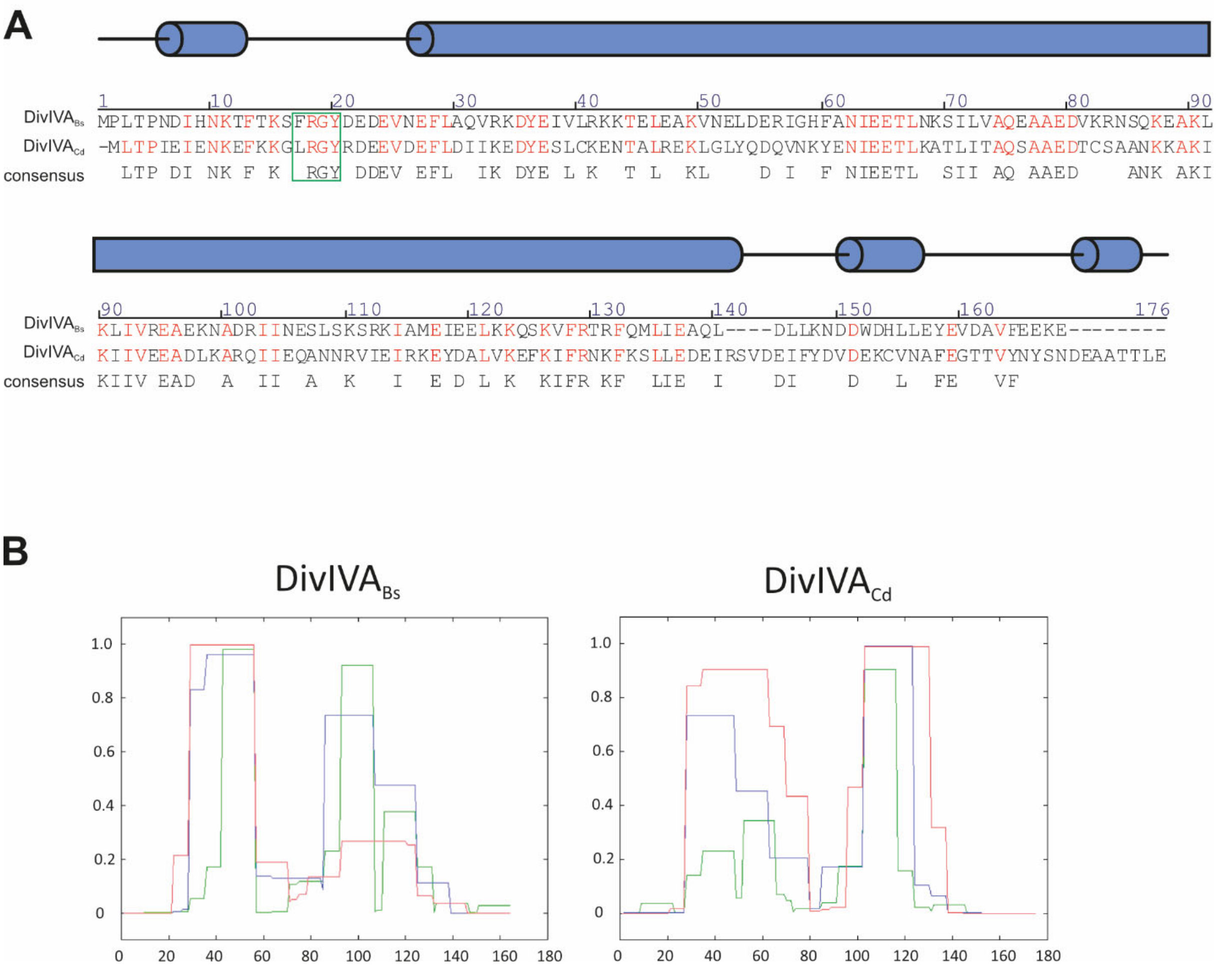

Fig. S1 Comparison of DivIVA$_{Bs}$ and DivIVA$_{Cd}$ primary structures and secondary structure and coiled-coils predictions

A. Secondary structure prediction and alignment of DivIVA$_{Cd}$ and DivIVA$_{Bs}$. DivIVA$_{Cd}$ and DivIVA$_{Bs}$ share 46.6% similarity and 31.1% identity. The residues, which are important for DivIVA$_{Bs}$ binding to the membrane and corresponding residues in DivIVA$_{Cd}$ are in green rectangle, identical residues are in red font.

B. Coiled-coil predictions. Green line corresponds to window of 14, blue line to window of 21, and red line to window of 28 residues. Predicted in COILS version 2.2 [5,6].

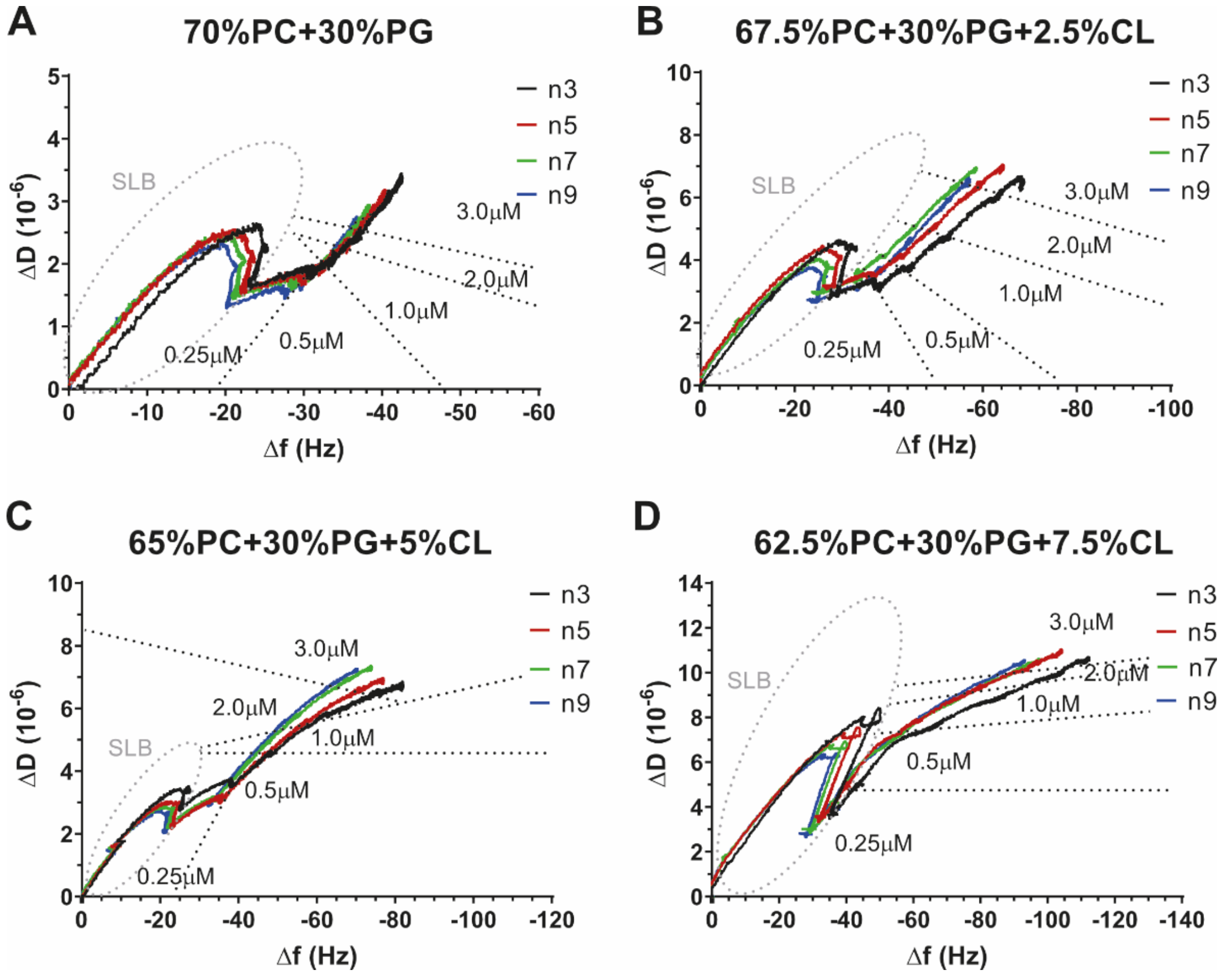

Fig. S2 Dynamic analysis of DivIVA$_{Cd}$ lipid binding to SLB

A-D. Plots of Δf against ΔD indicate that DivIVA$_{Cd}$ binding changes the morphology of the lipid bilayer. Representative measurements of the third (in black), fifth (in red), seventh (in green) and ninth (in blue) overtones as offset to zero after obtaining the baseline prior SLB formation are shown. The SLB formation is depicted in grey dashed oval, the plot is divided to areas corresponding to particular concentrations of DivIVA$_{Cd}$ by black dashed lines. The x-axis is reversed thus ascending curve indicates increased mass absorption. In the presence of cardiolipin and DivIVA$_{Cd}$ the curves are not overlapping, which means soft biofilm is formed.

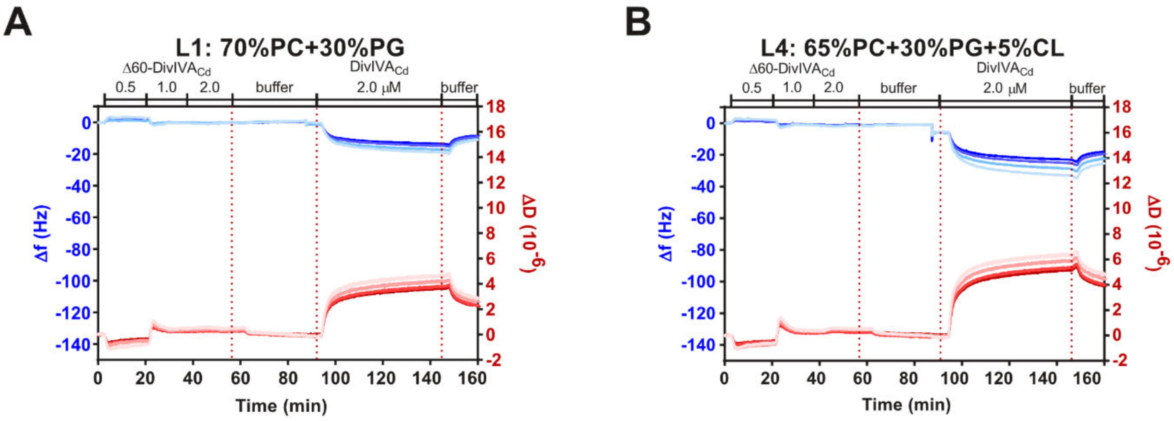

Fig. S3 QCM-D with N-terminally truncated DivIVA$_{Cd}$

The plots show details of Δf (in blue) and ΔD (in red) of the third, fifth, seventh and ninth overtone (lower overtones are in lighter tone, highest overtone is darkest), which were normalized to baseline obtained after SLB formation. As indicated above the plots, QCM-D measurements obtained after addition of 0.5µM Δ60-DivIVA$_{Cd}$, 1.0 µM Δ60-DivIVA$_{Cd}$, and 2.0 µM Δ60-DivIVA$_{Cd}$ to SLBs mixtures (A) L1 (70% PC and 30% PG) and (B) L4 (65% PC, 30% PG and 5% CL) are shown. As a positive control, in both experiments, after an extensive buffer wash (marked by the first two red dashed lines), we injected 2 µM full-length DivIVA$_{Cd}$ and we observed similar Δf response as in previous experiments with 2µM DivIVA$_{Cd}$ and L1 or L4 lipid mixture. The third dashed red line marks the final buffer rinse. While full-length DivIVA$_{Cd}$ produced a response similar to that seen previously under the same conditions, the Δ60-DivIVA$_{Cd}$ protein did not bind to either of the tested SLBs.

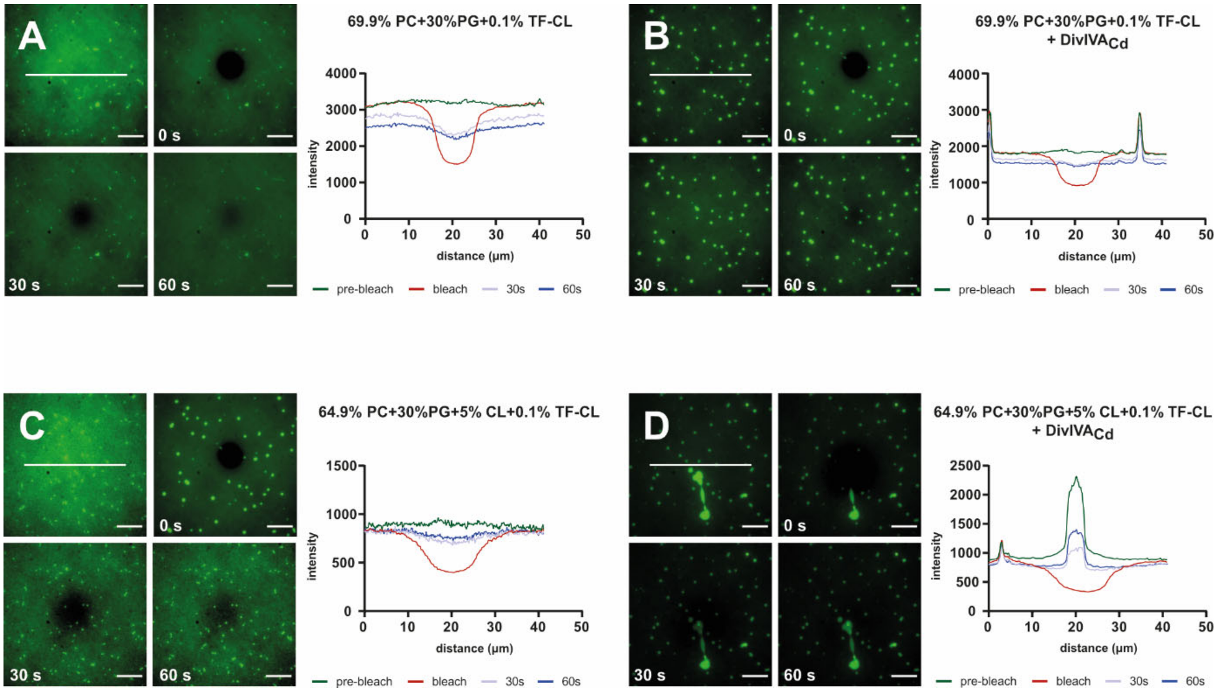

Fig. S4 FRAP revealed that lipid mobility is preserved in SLBs

SLBs composed of (A, B) 69.9% PC, 30% PG and 0.1% TF-CL (L1*) and (C, D) 64.9% PC, 30% PG+5% CL and 0.1% TF-CL (L4*) were subjected to FRAP analysis both before and after the addition of 2µM DivIVA$_{Cd}$. Representative images are shown with 10 µm scale bars. The plots represent fluorescence intensities before bleaching (green line), bleaching (red line), and 30 s and 60 s after bleaching (blue lines) measured along the white line shown in the upper left panel. The recovery within bleached areas indicate that the lipids remained dynamic within the SLBs even after DivIVA$_{Cd}$ addition and cluster formation.

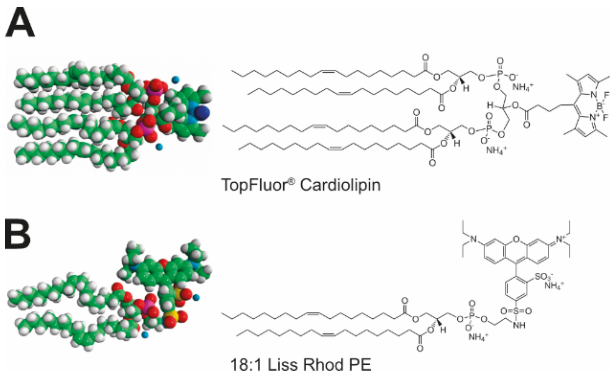

Fig. S5 Structures of fluorescent derivatives of CL and PE used in the study

(A) TopFluor® Cardiolipin: 1,1',2,2'-tetraoleoyl cardiolipin[4-(dipyrrometheneboron difluoride)butanoyl]; (B) 18:1 Liss Rhod PE: 1,2-dioleoyl-sn-glycero-3-phosphoethanolamine-N-(lissamine rhodamine B sulfonyl). Source: avantilipids.com

REFERENCES

1. Bachmann, B.J. Pedigrees of some mutant strains of Escherichia coli K-12. *Bacteriological Reviews* **1972**, *36*, 525–557, doi:10.1128/br.36.4.525-557.1972.

2. Taylor, R.G.; Walker, D.C.; Mclnnes, R.R. E.coli host strains significantly affect the quality of small scale plasmid DNA preparations used for sequencing. *Nucleic Acids Research* **1993**, *21*, 1677–1678, doi:https://doi.org/10.1093/nar/21.7.1677.

3. Studier, F.W.; Moffatt, B.A. Use of bacteriophage T7 RNA polymerase to direct selective high-level expression of cloned genes. *Journal of Molecular Biology* **1986**, *189*, 113–130, doi:10.1016/0022-2836(86)90385-2.

4. Bendezú, F.O.; De Boer, P.A.J. Conditional lethality, division defects, membrane involution, and endocytosis in mre and mrd shape mutants of Escherichia coli. *Journal of Bacteriology* **2008**, *190*, 1792–1811, doi:10.1128/JB.01322-07.

5. Combet, C.; Blanchet, C.; Geourjon, C.; Deléage, G. NPS@: Network protein sequence analysis. *Trends in Biochemical Sciences* **2000**, *25*, 147–150, doi:10.1016/s0968-0004(99)01540-6.

6. Lupas, A.; Van Dyke, M.; Stock, J. Predicting coiled coils from protein sequences. *Science* **1991**, *252*, 1162–1164, doi:10.1126/science.252.5009.1162.
